# Single opsin driven white noise ERGs in mice

**DOI:** 10.3389/fnins.2023.1211329

**Published:** 2023-07-31

**Authors:** Nina Stallwitz, Anneka Joachimsthaler, Jan Kremers

**Affiliations:** ^1^Department of Ophthalmology, University Hospital Erlangen, Erlangen, Germany; ^2^Animal Physiology, Department of Biology, Friedrich-Alexander-University Erlangen-Nürnberg, Erlangen, Germany

**Keywords:** electroretinography (ERG), mouse retina, photopigment, silent substitution, temporal white noise (TWN)

## Abstract

**Purpose:**

Electroretinograms elicited by photopigment isolating white noise stimuli (wnERGs) in mice were measured. The dependency of rod- and cone-opsin-driven wnERGs on mean luminance was studied.

**Methods:**

Temporal white noise stimuli (containing all frequencies up to 20 Hz, equal amplitudes, random phases) that modulated either rhodopsin, S-opsin or L*-opsin, using the double silent substitution technique, were used to record wnERGs in mice expressing a human L*-opsin instead of the native murine M-opsin. Responses were recorded at 4 mean luminances (MLs).

Impulse response functions (IRFs) were obtained by cross-correlating the wnERG recordings with the corresponding modulation of the photopigment excitation elicited by the stimulus. So-called modulation transfer functions (MTFs) were obtained by performing a Fourier transform on the IRFs.

Potentials of two repeated wnERG recordings at corresponding time points were plotted against each other. The correlation coefficient (r^2^_repr_) of the linear regression through these data was used to quantify reproducibility. Another correlation coefficient (r^2^_ML_) was used to quantify the correlations of the wnERGs obtained at different MLs with those at the highest (for cone isolating stimuli) or lowest (for rod isolating stimuli) ML.

**Results:**

IRFs showed an initial negative (a-wave like) trough N1 and a subsequent positive (b-wave like) peak P1. No oscillatory potential-like components were observed. At 0.4 and 1.0 log cd/m^2^ ML robust L*- and S-opsin-driven IRFs were obtained that displayed similar latencies and dependencies on ML. L*-opsin-driven IRFs were 2.5–3 times larger than S-opsin-driven IRFs. Rhodopsin-driven IRFs were observed at −0.8 and − 0.2 log cd/m^2^ and decreased in amplitude with increasing ML. They displayed an additional pronounced late negativity (N2), which may be a correlate of retinal ganglion cell activity.

R^2^_repr_ and r^2^_ML_ values increased for cones with increasing ML whereas they decreased for rods. For rhodopsin-driven MTFs at low MLs and L*-opsin-driven MTFs at high MLs amplitudes decreased with increasing frequency, with much faster decreasing amplitudes for rhodopsin. A delay was calculated from MTF phases showing larger delays for rhodopsin- vs. low delays for L*-opsin-driven responses.

**Conclusion:**

Opsin-isolating wnERGs in mice show characteristics of different retinal cell types and their connected pathways.

## 1. Introduction

The temporal white noise (TWN) stimulus can be an efficient way of characterizing physiological properties of responding systems (Marmarelis, 1978). In vision research, this stimulus has been extensively used in recordings from single neurons (Chichilnisky, 2001; Field et al., 2010). The TWN stimulus has been recently introduced in ERG measurements, to characterize ERG generating mechanisms in human subjects (Saul and Still, 2016; Zele et al., 2017; Adhikari et al., 2019), monkeys (Kremers et al., 2022) and mice (Wang et al., 2019; Stallwitz et al., 2022).

The TWN stimulus contains changes in luminance and/or chromaticity that are comparable with those in natural scenes so that the retina is kept in a physiological mode of operation. The cross correlation between response and stimulus results in the so-called impulse response function (IRF), which is the linear approximation of the system’s response to a flash. The Fourier transform of the IRF results in the modulation transfer function (MTF), describing the response of the system to sinewave stimuli of different temporal frequencies. However, the retina cannot be considered to be linear. ERGs are often measured to strong flashes in which a large amount of energy is compressed in a short time. As a result, the retina may be outside of the normal physiological mode of operation and its response may contain strong nonlinearities, that may result in substantial differences between the IRF and the flash ERG. The advantage of the TWN stimulus is therefore that the retina can be physiologically characterized when it is optimally functioning for transmitting visual information. A further advantage of the TWN stimulus is that it can be combined with the silent substitution technique and thus can isolate the responses of single photoreceptor types without changing the state of adaptation, thereby allowing comparisons of the results with different photoreceptor isolating stimuli (Kremers et al., 2022). Briefly, by modulating the luminance of light sources with different emission spectra with identical waveforms but different contrasts, all pigments, except one, can be silenced so that their excitations are not modulated (Kremers, 2003). The silent substitution technique is based on the distinct absorption spectra of the different photopigments. To obtain a sufficient modulation in excitation of the respective photopigments with the silent substitution technique, the absorption spectra of the photopigments should not overlap too strongly. This is warranted in humans and macaque monkeys. In mice, however, the absorption spectra of rhodopsin and middle wavelength sensitive (M-) pigments strongly overlap making the silent substitution technique less effective. [In the literature, the expression “photoreceptor response” is often used as synonymous to “photopigment excitation.” This is allowed for those cases where a photoreceptor contains only one type of photopigment and when there is no feedback of other photoreceptor types. The response of the photoreceptor is then exclusively determined by the excitation in the photopigment. This may not always be the case (Endeman et al., 2013). Mouse cones often express two different photopigment types - M- and S-opsin – (Lyubarsky et al., 1999; Applebury et al., 2000) so that their response is determined by the excitation of both pigments. The silent substitution method only considers pigment excitation. If a stimulus is silence for one pigment it may not be silence for the other pigment and therefore not for the concerning cone.] In the native murine retina, the rods have an absorption spectrum with a maximum at about 500 nm. Cones contain S- and M-opsins with absorption spectra that are maximal at 360 nm and 508 nm., respectively. In Opn1lw^LIAIS^ mice, the native M-pigment is replaced by the human L-pigment (henceforth called L*-opsin) with a maximal absorption at 561 nm. As a result, the spectral separation with rhodopsin is increased from about 8 nm (with the native M-opsin) to about 61 nm (with the L*-opsin). A detailed description of the LIAIS mouse (Greenwald et al., 2014) and of the usage of silent substitution stimuli in LIAIS mice can be found in previous publications (Tsai et al., 2015, 2017; Joachimsthaler and Kremers, 2019).

We previously investigated the ERG responses elicited by luminance TWN stimuli (white noise ERGs; wnERGs) in LIAIS mice (Stallwitz et al., 2022). We studied the dependency of the IRFs and MTFs on mean luminance. We also studied the correlations between wnERGs obtained at identical measurements, describing the reproducibility of the wnERGs, and at different MLs, giving information about the underlying ERG generating mechanisms (Stallwitz et al., 2022). In the present study, we extended these measurements by using photopigment-isolating TWN stimuli. Thus, in contrast to the previous study where rods and cones were stimulated simultaneously, we now stimulated them separately. WnERGs, IRFs and MTFs to single photopigment isolating TWN stimuli were obtained and analyzed, thereby giving additional insights to the contribution of rod- and cone-driven signals. In addition, reproducibility was studied by comparing ERG waveforms in two repeated measurements. The underlying ERG generating mechanisms at different MLs were studied by comparing the waveforms at different MLs with each other.

## 2. Materials and methods

### 2.1. Animals

All animal experiments were performed in accordance with the principles regarding the care and use of animals adopted by the Association for Research in Vision and Ophthalmology (ARVO). The conductance of these experiments was approved by the local ethics authorities (Regierungspräsidium Mittelfranken, Ansbach, Germany). The ERG measurements were performed on Opn1lw^LIAIS^ (LIAIS) mice which have a C57BL/6 J background. The mutant mice were created in the lab of Profs. Maureen and Jay Neitz from the University of Washington [Seattle, WA, United States; Greenwald et al., 2014] from whom we thankfully could obtain them. They were housed and bred in the Transgenic Mouse Facility in Erlangen, Germany, where they were kept in a 12 h light-12 h dark cycle with water and food available *ad libitum*.

Mice of the LIAIS strain express a human L-opsin variant instead of the native murine M-opsin, resulting in a 53 nm shift of spectral sensitivity of L*-opsins toward longer wavelengths from 508 nm to 561 nm (Jacobs et al., 1991; Sun et al., 1997; Lyubarsky et al., 1999) with no impact on the structure and function of these cones (Greenwald et al., 2014; Tsai et al., 2015; Joachimsthaler et al., 2017). The name LIAIS is based on the amino acids leucine, isoleucine, alanine, isoleucine and serine on positions 153, 171, 174, 178, and 180 of the L*-opsin variant. These locations are important in determining the spectral properties of the photopigment. As the gene for the M-opsin is located on the X chromosome, either hemizygous males or homozygous females have the L*-pigment and no native M-pigment. LIAIS mice express the endogenous murine S-opsin and rhodopsin in addition to the L*-opsin.

The recordings were performed on the same individual animals that were used in recording sessions for experiments with luminance TWN stimuli, the results of which are described in our previously published study (Stallwitz et al., 2022). Briefly, in total 11 hemizygous male LIAIS mice at an age between 14 and 20 weeks (mean: 16.35 ± 1.69 weeks of age) were used for recordings. Recordings were performed in separate sessions (either luminance, rhodopsin-, S-opsin- or L*-opsin-isolating stimuli) that were at least 1 week apart. Intrinsic noise recordings were performed on five additional hemizygous male LIAIS mice (13.14 weeks ±0.35 of age).

### 2.2. Preparation

The mice were dark adapted overnight. All further handling was performed under dim red light. Because of the L*-opsin, cone responses of LIAIS might be affected by the red light. However, pilot studies showed that there were no differences between responses of WT and LIAIS mice performed after red light or infra-red preparation (Stüwe, Stallwitz, Kremers and Joachimsthaler, unpublished data).

A mixture of 50:10 mg/kg ketamine/xylazine (Ketavet; Pfizer, Karlsruhe, Germany; Rompun 2%; Bayer AG, Leverkusen, Germany) was injected intramuscularly to anaesthetize the animals. By applying drops of tropicamide (Mydriaticum Stulln, 5 mg/mL; Pharma Stulln, Stulln, Germany) and phenylephrine-hydrochloride (Neosynephrin POS 5%; Ursapharm, Saarbrücken, Germany) topically, the pupils of the animals were dilated. A subcutaneous injection of 400 μL saline (0.9%) prior to the recordings prevented the animals from dehydrating while being under anesthesia. The animals were placed on a heated platform during ERG recordings, ensuring maintenance of body temperature. A needle placed subcutaneously at the base of the tail served as ground electrode, while another needle placed subcutaneously and medially to the ears served as reference electrode. Active contact lens electrodes (Diagnosys LLC, Cambridge, United Kingdom) were filled with Corneregel (Dr. Mann Pharma, Berlin, Germany). The electrodes were connected to fibers through which the stimuli were applied.

### 2.3. TWN stimuli

The detailed description of TWN stimulus can be found in our previous publication (Stallwitz et al., 2022). Briefly, the TWN stimuli were created by modulating the luminance outputs of three differently colored light emitting diodes (LEDs). Each LED was modulated around a mean luminance. The stimulus was generated by the full field light guide electrodes of the setup (Diagnosys LLC, Cambridge, United Kingdom) and the Espion software (Diagnosys LLC, Cambridge, United Kingdom) controlled the stimulation. The stimulus was calculated by performing an inverse Fourier transform of the stimulus in the frequency domain with equal amplitudes of integer frequencies up to 20 Hz and with random phases at each frequency [see Figure 1 in Zele et al. (2017)]. No Frequencies above 20 Hz were included in the stimulus, because ERG responses to these frequencies are very small in mice (Tsai et al., 2015¸ 2017) and therefore barely contribute to the ERG.

**Figure 1:**
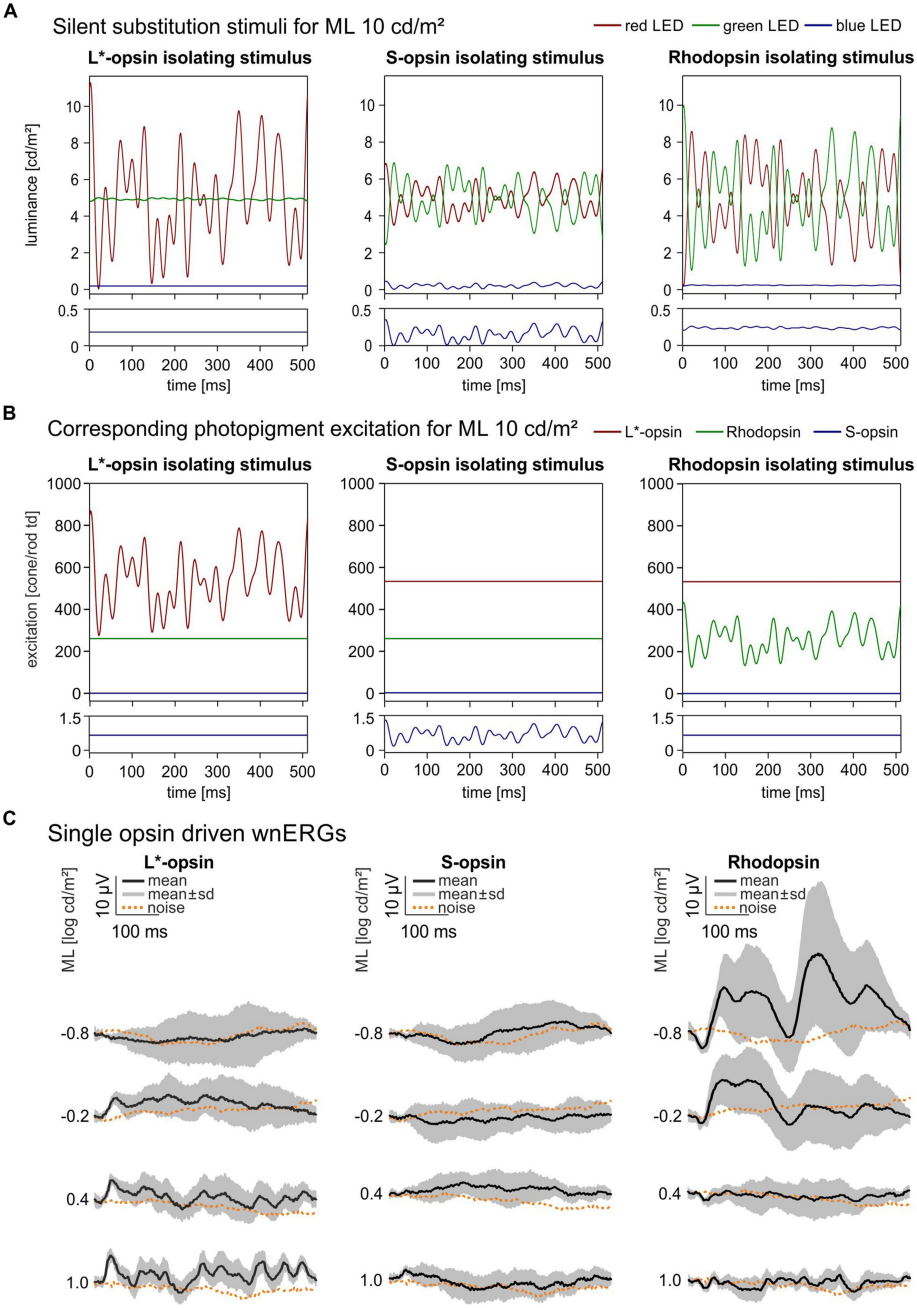
Single opsin driven wnERGs in mice. **(A)** Single opsin isolating Temporal White Noise stimuli for L*-opsin, S-opsin and rhodopsin shown for 1 ML. TWN stimuli are generated by three LEDs (red, green, blue). The luminance output of the LEDs are given as a function of time. The lower plots are enlargements of the blue LED outputs. **(B)** Photopigment excitation as a function of time for the three silent substitution TWN stimuli. The lower plots are enlargements of the S-opsin excitations. The excitation of only one pigment is modulated. Furthermore, the modulation form is identical in the three conditions (although the contrast, i.e., the modulation normalized to the mean excitation, differ in the three conditions; see text). Finally, the mean excitations are identical in the three conditions, indicating that the states of adaptation are identical in the three conditions. **(C)** Resulting single opsin driven wnERGs to silent substitution TWN stimuli for L*-opsin, S-opsin and rhodopsin at four different MLs. Black traces represent mean wnERGs averaged across all animals, gray areas indicate standard deviations of the measurements obtained from the different animals. Corresponding noise measurements for each ML are shown as dotted orange lines.

In the present study, the LEDs were modulated according to a double silent substitution to generate a TWN stimulus that isolated the responses of a single photopigment type (see Figure 1A for a description of the luminance modulation in each of the three LEDs). Please observe that the mean luminance of each LED was identical for the three pigment-isolating conditions. As a result, the ML and mean chromaticity were the same in each condition and the states of adaptation were also identical for all three conditions. An R:G:B luminance ratio of 32:32:1 was used resulting in the same mean chromaticity for all conditions. The ratio was chosen in order to optimize the stimulus strength for each of the three photopigments, determined by multiplying the pigment absorption spectra obtained, corrected for pre-retinal absorption, with the LED emission spectra and integrating over wavelength. An equivalent description of stimulus calculations in the macaque monkey was described previously (Kremers et al., 2022). The measurements were repeated at −0.8, −0.2, 0.4 and 1.0 log phot cd/m^2^ ML (i.e., 0.16, 0.62, 2.5 and 10 phot cd/m^2^; equal to −0.127, 0.461, 1.066 and 1.668 log scot cd/m^2^). Silent substitution TWN stimuli achieved maximal photopigment contrasts of 52, 48, and 77% for rhodopsin, L*-opsin and S-opsin, respectively. However, the mean S-opsin excitation was between 2 and 3 orders of magnitude weaker than in rhodopsin and L*-opsin (see Table 1 and Figure 1B). The pigment excitations are expressed as cone td or rod td, which expresses retinal irradiance weighted by pigment sensitivity in the human eye. The quantification is only valid for the mouse eye when assuming that it is isometric with (i.e., a scaled down version of) the human eye and when the pigment concentrations in the two eyes are identical. Deviations from these assumptions may lead to differences in irradiance in absolute but not in relative terms. A schematic description of the mouse eye was made previously (Remtulla and Hallett, 1985). The mean pigment excitations are identical in the three pigment isolating conditions, indicating that the retina was in an identical state of adaptation, thus enabling comparison of the results obtained in these conditions. Two measurements with 300 sweeps each (512 ms stimulation per sweep) were recorded at all ML levels. These two recordings were used to analyze the reproducibility of the recordings. For further analysis both recordings were averaged.

**Table 1:** Silent substitution stimuli settings at the highest ML.

LED	ML Ratio	LED Contrasts		Mean excitation (Cone or rod td)	Cone or rod Contrasts
S-opsin isolating conditions					
Red	32	29.8495795	S-opsin	0.71	76.98
Green	32	−39.632953^a^	L*-opsin	533.31	0
Blue	1	100	Rhodopsin	260.67	0
L*-opsin isolating conditions					
Red	32	100	S-opsin	0.71	0
Green	32	−1.75725555^a^	L*-opsin	533.31	48.24
Blue	1	0.34686075	Rhodopsin	260.67	0
Rhodopsin isolating conditions					
Red	32	−75.3197884^a^	S-opsin	0.71	0
Green	32	80	L*-opsin	533.31	0
Blue	1	−15.7952492^a^	Rhodopsin	260.67	51.57

a A negative contrast represents a stimulus with a mirror imaged TWN profile relative to those with a positive contrast (see Figure 1A). The pigment excitations have a positive contrast, showing that they all have the same temporal profile (i.e., are not mirror imaged relative to each other; see Figure 1B) and only differ in their contrast.

### 2.4. ERG recordings

In each animal, recordings were performed in four separate sessions: one session for luminance modulation as described in Stallwitz et al. (2022) and three sessions for opsin-isolating stimuli described in the present study. The sessions were at least 1 week apart so that the animals could fully recover from a previous recording. The recording period (3 weeks between first and last measurements) was sufficiently short to neglect age effects. In one session, the responses to TWN stimuli with the same spectral conditions (i.e., modulation of either luminance, rhodopsin-, S-opsin- or L*-opsin-excitation) were recorded. To further rule out remaining age effects, the sessions with the different pigment isolating conditions were randomized for each mouse. The animal was adapted for 1 min to the ML of the following stimulus before the recording session started. The first sweep (512 ms) of each recording was discarded to avoid onset artifacts. The protocols lasted between 20 and 30 min (for opsin-isolating conditions) and 50 to 60 min for luminance modulation after which the animals were allowed to wake up.

As described in our previous study (Stallwitz et al., 2022) intrinsic noise measurements were performed on an additional group of five male LIAIS mice. For intrinsic noise measurements ERG responses to a steady background at the same ML as the wnERGs were recorded. These noise measurements were compared with opsin isolating wnERGs (see above) and to obtain an estimate of signal-to-noise ratios. After 5 min of adaptation to the first shown ML, responses to each ML used for the wnERGs were measured. Again, for all MLs two measurements of 300 sweeps each were recorded. The protocol lasted around 50 min and the animals were allowed to wake up afterwards.

All ERG recordings were band-pass filtered with 0.125 Hz and 300 Hz cut-off frequencies. The signal was digitized and with a 1,000 Hz sampling frequency.

### 2.5. Data analysis

#### 2.5.1. Reproducibility and correlations at different mean luminances

To investigate the reproducibility of the wnERGs, the 1st recordings of all animals were averaged at each ML and each pigment isolating condition and the potentials at each time point were plotted against the averaged potentials at identical time points obtained during the 2nd recordings. From the linear regressions through each plot, the correlation coefficients, r^2^_repr_, quantified the reproducibility of the recordings. In the present case, the correlation coefficient varied between 0 and 1, whereby 0 indicated no concordance of both measurements and 1 implied complete reproducibility of the two recordings.

The underlying mechanisms of recordings at different MLs were studied by further averaging the 1st and 2nd recordings of all animals at each stimulus condition and plotting the potentials at the concerning ML as a function of the potentials at identical time points obtained at the highest (for cones) and the lowest (for rods) ML. The linear regression through the plots gave another correlation coefficient r^2^_ML_, quantifying the contribution of cone- and rod-signals at each ML.

#### 2.5.2. Impulse response functions

**Figure 2:**
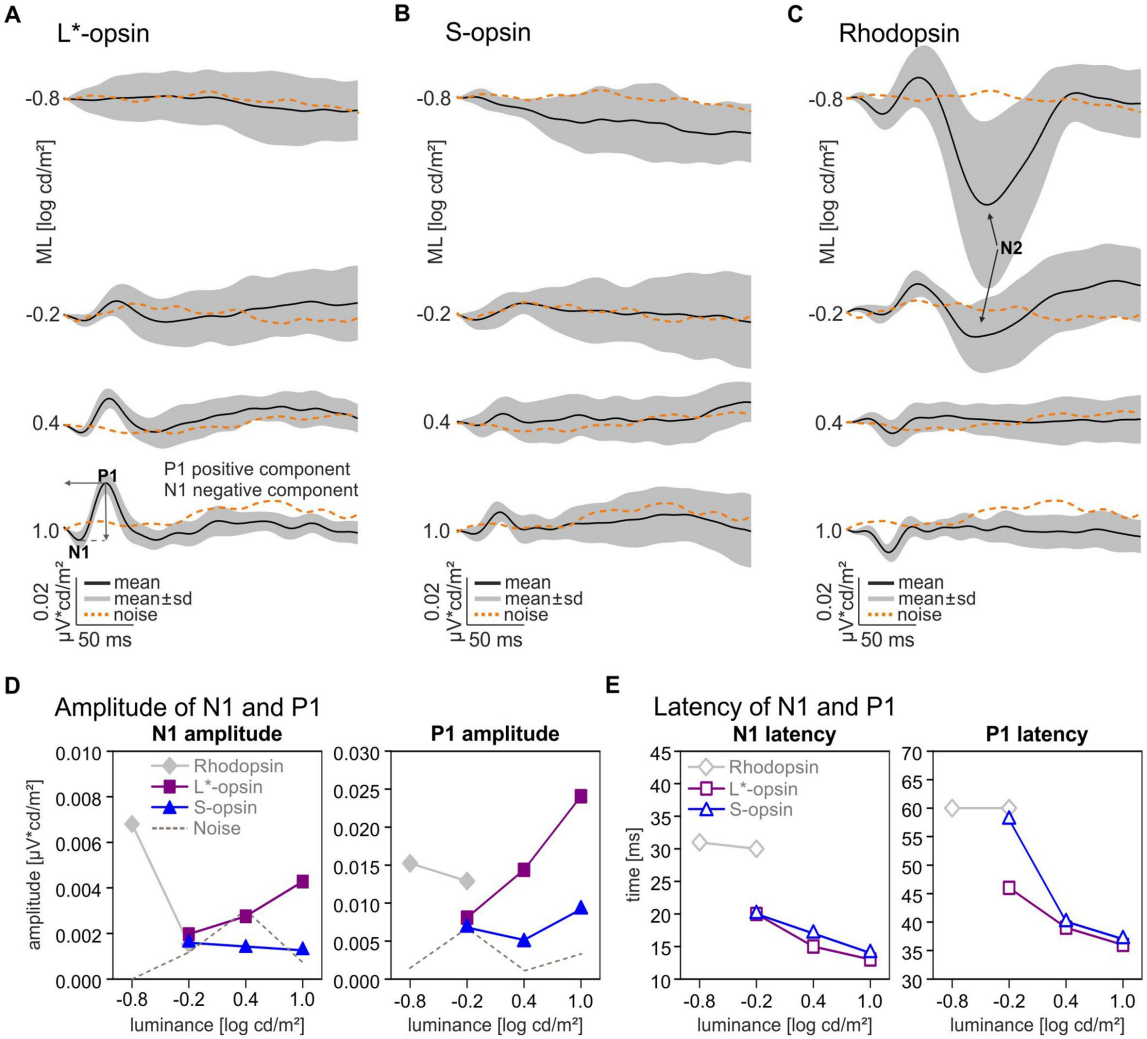
Single opsin driven Impulse Response Functions and components. Averaged Impulse Response Functions driven by L*-opsin **(A)**, S-opsin **(B)**, and rhodopsin **(C)** at four different MLs (black lines) and averaged intrinsic noise signals (dashed orange lines). The gray areas indicate standard deviations. Wave components: first negative trough: – N1, first positive peak: P1; second negative trough for rhodopsin-driven wnERGs: N2, (arrows). **(D)** N1 and P1 Amplitudes and **(E)** latencies for L*-opsin (magenta squares), S-opsin (blue triangles), rhodopsin (gray diamonds).

The Impulse Response Function (IRF) was obtained by cross correlating the averaged wnERGs at each ML with the corresponding photoreceptor excitations as displayed in Figure 1B. Therefore, each wnERG result was multiplied with the photoreceptor excitation at each time stamp of the stimulus and summed for all 512 timestamps. This resulted in the cross-correlation at *t* = 0 ms. Then, the wnERG was shifted by 1 ms and cross-correlated again with the stimulus. The procedure was repeated at in total 257 time points between 0 and 256 ms. The cross correlation as a function of the time shift results in the IRF. A more detailed description can be found elsewhere (Zele et al., 2017). The resultant IRFs are shown in Figure 2. The IRFs display two prominent wave components for all MLs: an initial negative – N1 – and a following positive – P1 – going component. An additional second negative trough N2 after P1 at the lowest MLs can be observed with the rhodopsin-isolating stimuli. The difference between the baseline (defined as mean of the first 6 ms of recording) and the first and the second negative trough defined the amplitude of N1 and N2, respectively. The difference between the first negative trough N1 and the following positive peak defined the amplitude of P1. The latencies of the negative and positive components were defined as the time from *t* = 0 (no time shift) to the time shifts at the troughs or peak.

The intrinsic noise measurements were analyzed in an identical manner as the wnERGs. The definition of a meaningful response or IRF component was an amplitude in the wnERG or the IRF component that was larger than the noise response or component.

#### 2.5.3. Modulation transfer function

The Modulation Transfer Function (MTF) was obtained by Fourier transform of the IRF. The MTFs gave the response amplitudes and phases as a function of temporal frequency. They are equal to the amplitudes and phases of the linear approximation to sinewave stimuli with the same temporal frequency. Again, a comparison with actually measured responses to sinewave stimuli can give an indication about the involved nonlinearities.

## 3. Results

### 3.1. wnERGs to opsin-isolating TWN stimuli

Figure 1A displays the LED luminances as a function of time during a sweep for each single opsin isolating TWN stimulus. Figure 1B shows the corresponding photopigment excitations as a function of time. The lower plots are enlargements of the S-opsin excitation. Clearly, S-opsin modulation was only achieved with S-opsin isolating stimuli. However, the mean excitation was about 750 times weaker than the mean L*-opsin excitation and about 365 times weaker than the mean rhodopsin excitation (see Table 1). The drawn curves in Figure 1C are the resulting wnERGs at four different MLs between −0.8 log cd/m^2^ (mesopic) and 1.0 log cd/m^2^ (low photopic) ML. The data for the L*-opsin-, S-opsin- or rhodopsin-isolating conditions are shown in the left, middle and right columns, respectively. The dotted curves are the noise recordings at the corresponding conditions. Cone-driven wnERGs could not be obtained at a ML of 0.8 log cd/m^2^. L*-opsin-driven wnERGs exceeded noise for all other MLs and their responses increased with increasing ML. S-opsin-driven wnERGs were hardly visible and hardly exceeded noise even at the highest ML. These results are not surprising in face of the abovementioned weak S-opsin mean excitation. In contrast, rhodopsin-driven wnERGs showed strongest responses at the lowest MLs (Figure 1C right) and the response decreased to noise level with increasing ML. In agreement with previous findings (Stallwitz et al., 2022), L*-opsin- and rhodopsin-driven wnERG waveforms are clearly different.

### 3.2. Rod- and cone-driven IRFs

The single opsin driven wnERGs were cross-correlated with the corresponding photoreceptor excitation to obtain the photopigment specific IRFs (Figure 2). In agreement with previous results (Zele et al., 2017; Kremers et al., 2022; Stallwitz et al., 2022), the IRFs roughly resembled flash ERGs but lacked components that resembled oscillatory potentials. Measurable L*- and S-opsin-driven IRFs were obtained at −0.2 log cd/m^2^ ML and higher (Figures 2A,B). Particularly for the lowest ML and for S-opsin-driven IRFs the amplitudes barely exceeded the values obtained after applying the same procedure on noise measurements; however, the latencies of the components fitted with the expected timing so that they were considered to be significant. With increasing ML from −0.2 log cd/m^2^ to 1.0 log cd/m^2^, the N1 amplitude of the L*-opsin-driven IRF increased by a factor of 2.17 whereas the P1 amplitude increased by a factor of 2.97 (Figure 2D, filled magenta squares). The N1 latencies decreased from about 20 to 13 ms and P1 latencies decreased from about 47 to 37 ms at this ML increase (Figure 2E, open magenta squares). The amplitudes for S-opsin-driven IRFs remained relatively constant with increasing ML (Figure 2D, closed blue triangles), but the responses were also barely above noise level. The latencies decreased with increasing ML from 20 to 14 ms for N1 and from 58 to 37 ms for P1 (Figure 2E, open blue triangles). The IRFs at −0.2 log cd/m^2^ ML are indicative for some rod intrusion in the P1. Maximal S-opsin-driven amplitudes for P1 were 2.62 times smaller than maximal L*-opsin-driven amplitudes despite the larger contrast used for the S-pigment (see Table 1). The N1 and P1 latencies were similar for L*- and S-pigment isolating stimuli although, as mentioned above, the P1 latency at −0.2 log cd/m^2^ ML indicates that rod responses may have intruded.

At the lowest ML of −0.8 log cd/m^2^ only rhodopsin-driven IRFs were above noise (Figure 2C). At 0.4 and 1.0 log cd/m^2^ ML, the rhodopsin-driven IRFs resembled the cone opsin-driven IRFs but with inverted polarity.

Rhodopsin-driven IRF components were largest at the lowest MLs and the amplitudes decreased with increasing ML (Figure 2D, gray diamonds). N1 and P1 latencies were about 32 ms (N1) and 60 ms (P1) for rhodopsin-driven IRFs at −0.8 and − 0.2 log cd/m^2^ ML (Figure 2E, open gray diamonds). The amplitudes and latencies at the two highest MLs are not shown in Figures 2D,E due to the abovementioned cone intrusion.

Rhodopsin-driven IRFs show a substantial additional late negative trough (N2) after the P1 (Figure 2C, marked with arrows). The component is only visible for the two lowest intensities with a latency of 122 ms at −0.8 log cd/m^2^ ML and of 111 ms at −0.2 log cd/m^2^ ML. This N2 component is remarkably large and its amplitude decreased from 0.045 μV*cd/m^2^ at −0.8 log cd/m^2^ ML to 0.011 μV*cd/m^2^ at −0.2 log cd/m^2^ ML.

**Figure 3:**
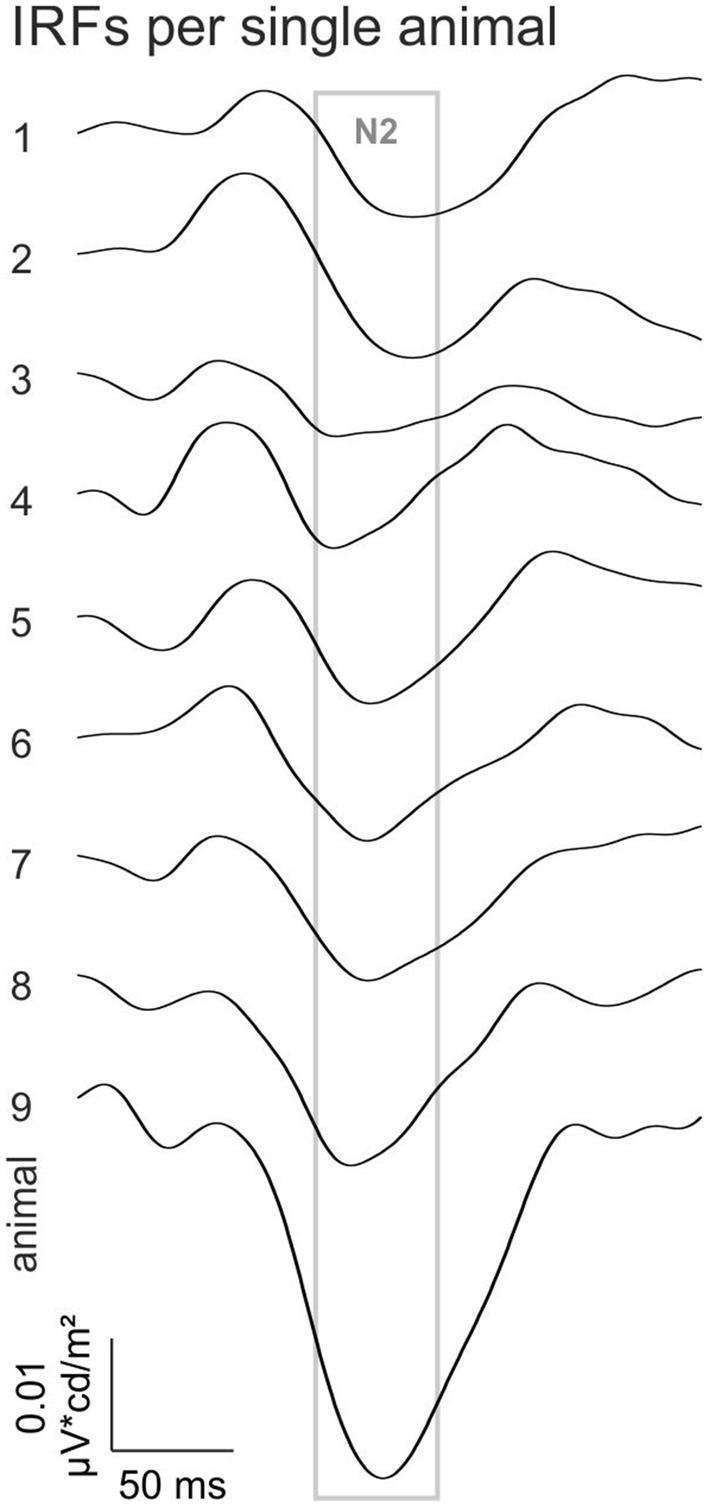
Rhodopsin-driven IRFs of the individual animals at −0.8 log cd/m^2^ ML. The gray square indicates the time window in which the N2 appeared.

To study if the N2 component is a general feature in all animals the individual IRFs at 0.8 log cd/m^2^ ML are shown in Figure 3. The N2 component is prominently present within an interval between 105 and 140 ms in the IRFs for all individual animals. The N2 therefore seems to be a reliable component of the rhodopsin-driven IRF.

### 3.3. Correlation coefficients

**Figure 4:**
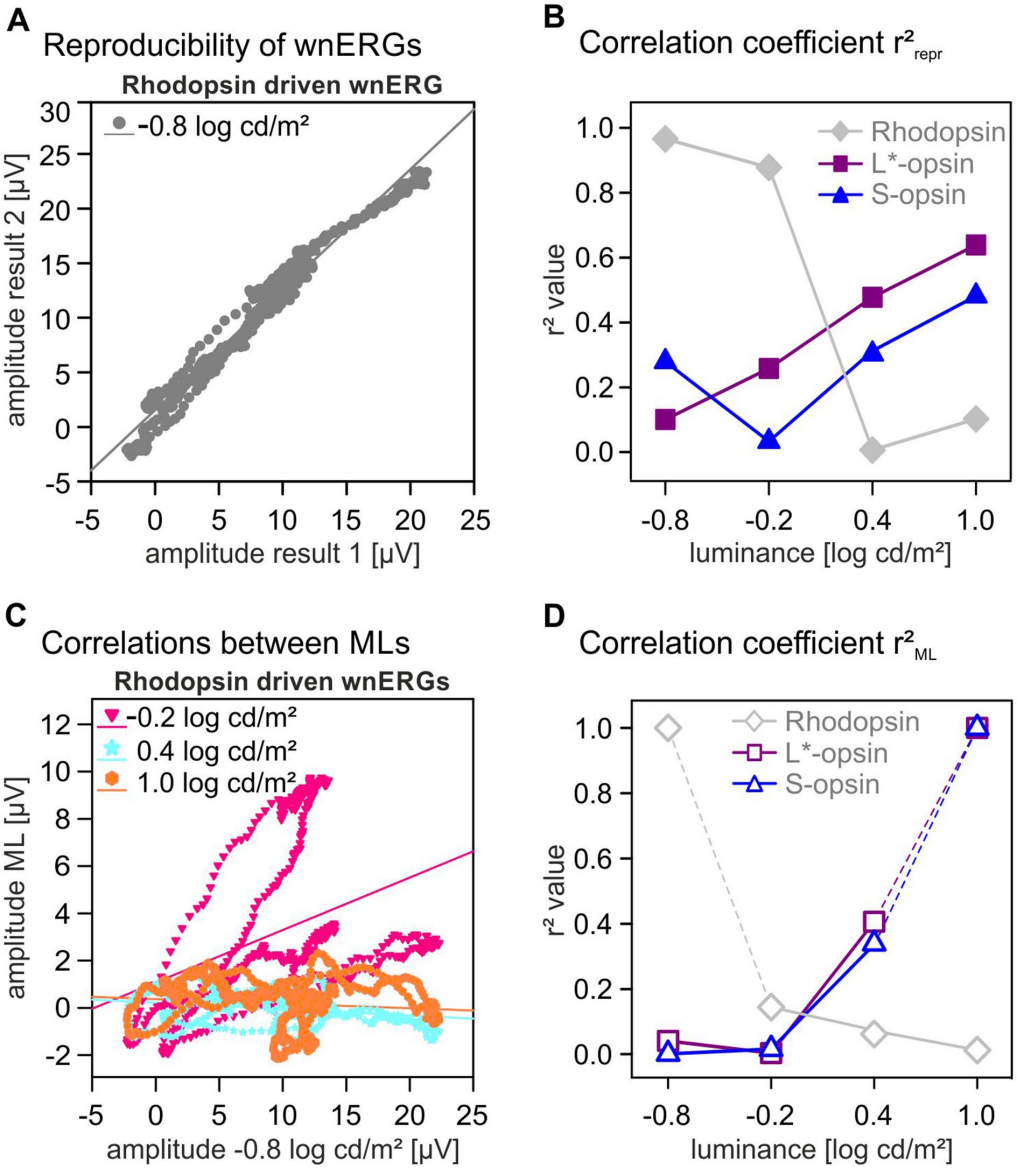
Correlation coefficients. **(A)** Potentials of two identical wnERG recordings are plotted against each other. From the linear regressions through the data, correlation coefficient r^2^_repr_ were obtained to quantify the reproducibility of measurements. **(B)** r^2^_repr_ correlation coefficients for different opsin isolating conditions (gray diamonds for rhodopsin, magenta squares for L*-opsin, blue triangles for S-opsin) given as a function of ML. **(C)** Plots of the potentials obtained from rhodopsin-driven wnERGs recordings as a function of the potentials obtained at −0.8 log cd/m^2^. From the linear regressions through the data correlation coefficient r^2^_ML_ were obtained that quantified the similarity of the signals at different MLs and thus also the similarity of the underlying ERG-generating mechanisms. **(D)** r^2^_ML_ values for all three opsin isolating conditions (gray diamonds for rhodopsin, magenta squares for L*-opsin, blue triangles for S-opsin) given as a function of ML. The values of rhodopsin-driven wnERGs at 0.8 log cd/m^2^ and of cone opsin-driven wnERGs at 1.0 log cd/m^2^ are, by definition, 1.0 because these are obtained from correlations between identical signals and thus not informative.

To study the wnERG waveforms in more detail, we analyzed their reproducibility. For each photopigment isolating condition and ML, two repeated measurements were performed. The 1^st^ and 2^nd^ wnERGs were each averaged across animals and the potentials at identical time points during stimulation were plotted against each other (see Figure 4A for the rhodopsin-driven wnERGs at 0.8 log cd/m^2^ ML). The correlation coefficients (r^2^_repr_) of the linear regressions through the data gave a quantification of the reproducibility. We have previously performed this analysis for luminance TWN stimuli in mice (Stallwitz et al., 2022) and for luminance and cone-driven wnERGs in macaques (Kremers et al., 2022). The correlation coefficients are plotted as a function of ML in Figure 4B for each photopigment isolating stimulus. Values of r^2^_repr_ for rhodopsin-driven wnERGs were maximal at low MLs where r^2^_repr_ was close to 1, indicating excellent reproducibility. r^2^_repr_ decreased with increasing ML to 0.006 at 0.4 log cd/m^2^, indicating an absence of reproducibility. L*-opsin-driven wnERGs showed increasing reproducibility with increasing ML from 0.1 at 0.8 log cd/m^2^ ML to 0.65 at 1.0 log cd/m^2^ ML. Reproducibility of S-opsin-driven wnERGs was minimal at −0.2 log cd/m^2^ ML and r^2^_repr_ had a maximal value of 0.45 at 1.0 log cd/m^2^ ML. r^2^_repr_ values were significant (*p* < 0.05) for all correlations except for rhodopsin at a ML of 0.4 log cd/m^2^ (*p* = 0.07).

As shown in Figure 1C, the L*-opsin- and rhodopsin-driven wnERG waveforms differed strongly. To study the mechanisms underlying the wnERGs, the results of the first and second measurements at each ML were averaged. We then obtained the correlation coefficient (r^2^_ML_) from the linear regressions of the plots of the potentials of the rhodopsin-driven wnERG at a given ML vs. the potentials at the lowest ML (see Figure 4C). The potentials of cone opsin-driven wnERGs at each ML were plotted against those obtained at the highest ML (see Supplemental material). Figure 4D shows the r^2^_ML_ values for rhodopsin, L*-opsin and S-opsin isolating stimuli. r^2^_ML_ values of rhodopsin-and opsin-driven wnERGs show a contrary dependency on ML: whereas r^2^_ML_ values decreased, from 0.15 to 0, with increasing ML for rhodopsin-driven ERGs, those of opsin-driven wnERGs increased with increasing ML from around 0 to about 0.35. Except for S-opsin at a ML of −0.8 log cd/m^2^ (*p* = 0.42) and L*-opsin at a ML of −0.2 log cd/m^2^ (*p* = 0.2) all correlations regarding r^2^_ML_ values were significant (*p* < 0.05). As mentioned above, L*-opsin- and rhodopsin-driven wnERGs differ strongly. To demonstrate the different mechanisms contributing to signals for L*-opsin- and rhodopsin-isolating stimulus rhodopsin-wnERGs at the lowest ML were correlated with L*-opsin-wnERGs at the highest ML (i.e., those conditions that resulted in the largest response). The resulting r^2^ value is 4.09 x 10^-6^ (*p* = 0.96), indicating no similarities between the two responses. In contrast, when correlating L*-opsin-wnERGs at the highest ML to S-opsin-wnERGs at the highest ML, the resulting r^2^ is 0.2 (*p* < 0.05), indicating moderate similarity between the two results.

In our previous study we suggested that wnERGs to luminance stimuli are rhodopsin-driven at low MLs and opsin-driven at high MLs (Stallwitz et al., 2022). Since the luminance wnERGs and single-opsin-driven wnERGs were measured within the same individual animals, we addressed this hypothesis by correlating luminance and photopigment-driven wnERGs. The correlation of rhodopsin-driven and luminance wnERGs at their lowest ML (−0.8 log cd/m^2^ for rhodopsin isolation and − 0.7 log cd/m^2^ for luminance modulation) had an r^2^ of 0.37 (*p* < 0.05), indicating moderate similarities between the two. Furthermore, the correlation between L*-opsin-driven and luminance wnERGs at the highest ML (1.0 log cd/m^2^ and 1.1 log cd/m^2^ for L*-opsin and luminance modulation, respectively) gave a r^2^ value of 0.69 (*p* < 0.05), proving that luminance wnERGs at high MLs were indeed L*-opsin-driven. We should stress that the photopigment isolating stimuli and the luminance stimuli had different chromaticities. This may have affected the correlations of the wnERGs obtained with the two conditions.

### 3.4. Modulation transfer function

MTFs represent the amplitudes and phases of the responses of a linear system’s approximation to sinewave stimuli as a function of the temporal frequency. They are obtained by performing a Fourier transform on the IRFs.

**Figure 5:**
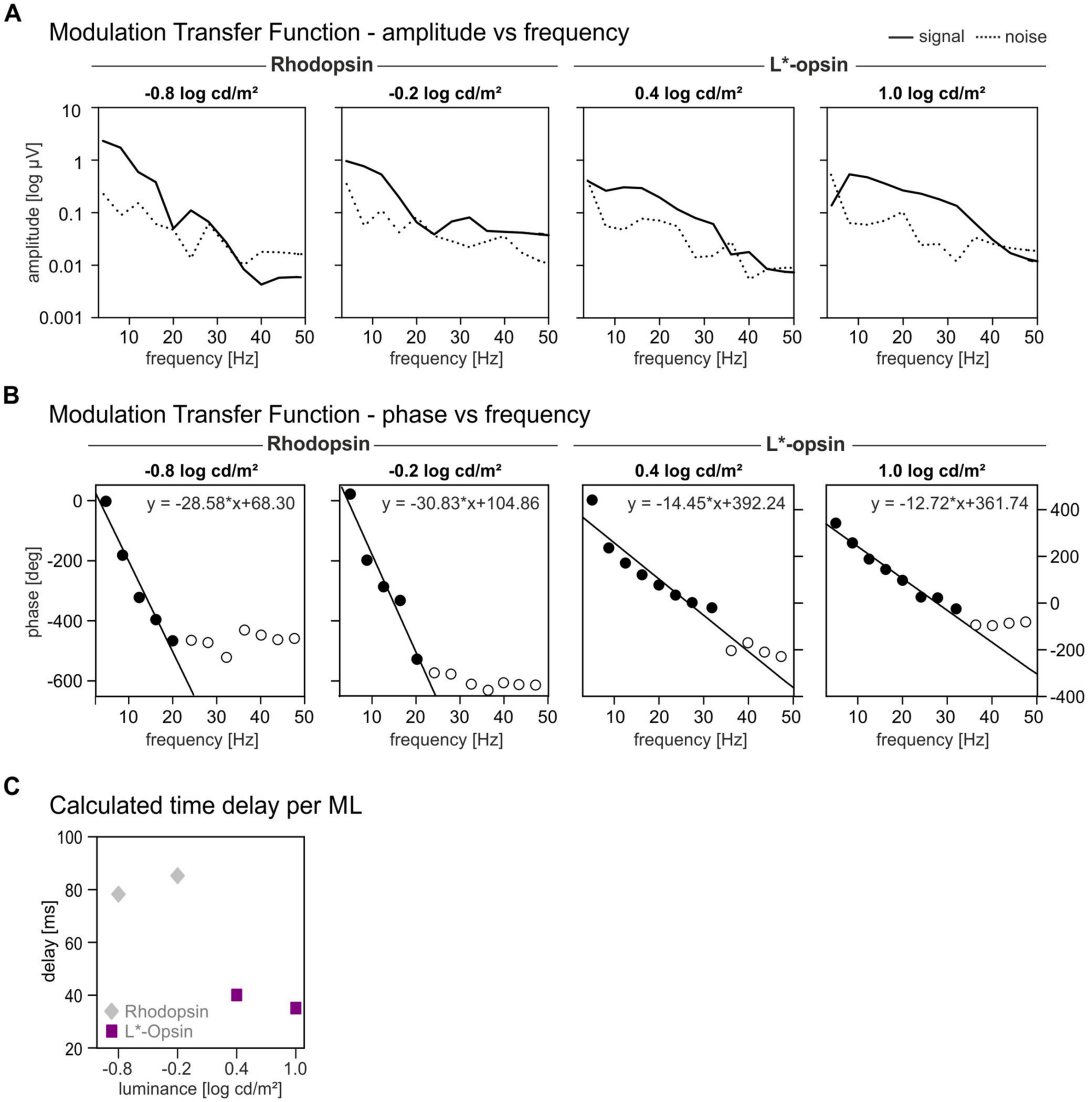
Modulation Transfer Functions for rhodopsin and L*-opsin driven wnERGs. Response amplitudes **(A)** and phases **(B)** as function of temporal frequency shown separately for different MLs. Rhodopsin-driven MTFs are only shown for the two lowest MLs whereas L*-opsin-driven MTFs are shown for the two highest MLs (as indicated above the graphs). Closed symbols indicate responses where the phases were used for linear regression. **(C)** Calculated time delays for rhodopsin (gray diamonds) and L*-opsin (magenta squares) at different MLs. The MTFs are based on the IRFs obtained from the averaged wnERGs.

Figure 5A presents the amplitudes of rhodopsin and L*-opsin-driven signals as a function of temporal frequency. We only show the MTFs of L*-opsin-driven responses at high MLs and of rhodopsin-driven responses at low MLs because their IRFs were significantly above noise. Rhodopsin-driven MTFs show a gradual amplitude decrease up to 20–30 Hz. Also shown are noise amplitudes obtained from Fourier transform on IRFs from noise wnERGs. It can be seen that the amplitudes are larger than noise for frequencies up to about 20 Hz. L*-opsin-driven MTFs at 0.4 log cd/m^2^ ML had amplitudes that were relatively constant between 7 and 17 Hz and decreased at higher frequencies. The amplitudes obtained at 1.0 log cd/m^2^ ML showed a band-pass characteristic with a maximum at about 7 Hz. The L*-opsin-driven responses were substantially larger than noise for frequencies up to about 30 Hz.

The phases of the MTFs are plotted vs. temporal frequency in Figure 5B. All phases are plotted but we used only the phases where the amplitudes were above noise (i.e., up to 20 Hz and 30 Hz for rhodopsin-and L*-cone-driven responses respectively; closed symbols) for further analysis. The open symbols are phases at frequencies where the SNR ratio was too small to be regarded as a reliable signal. These phases were excluded from further analysis. Phases of rhodopsin-driven MTFs decreased in an approximately linear manner up to a frequency of 20 Hz. Phases of L*-opsin-driven MTFs decreased also linearly up to 30 Hz. A linear relationship between phase vs. frequency plots indicates the presence of a fixed time delay in the responses. In that case, the slopes of the linear regressions (on average − 29.7 °/Hz for rod-driven MTFs and − 13.85 °/Hz for cone-driven MTFs) are proportional to the apparent delay time. The apparent delays are displayed as a function of ML in Figure 5C. The delays were about 82.5 ms for rhodopsin-driven responses and about 37.7 ms for L*-opsin-driven responses. Directly measured cone-driven MTFs, obtained from responses to sinewave stimuli, resulted in apparent delays between 33 and 42 ms (Tsai et al., 2015, 2017) which is very similar to the delays found here. On the other hand, rod-driven sinewave responses at low luminances had apparent delays between 40 and 53 ms (Tsai et al., 2017), which is substantially smaller than the delays found in the present study. We propose that this is caused by the large N2 component in the IRFs. An equivalent of this component may be absent in the responses to sinewave stimuli.

## 4. Discussion

The purpose of the present study was to describe the IRFs and MTFs driven by single photopigments in the mouse retina at different mean luminances. The main findings were: (1) The presence of a pronounced late component (N2) in the rhodopsin-driven IRFs, (2) The presence of rhodopsin-driven IRFs at high luminances that resemble inverted cone opsin-driven IRFs, (3) Comparisons with luminance-driven wnERGs and IRFs show similarities with rhodopsin-driven responses at low luminances and with cone opsin-driven responses at high luminances. We further compare the mouse IRFs and MTFs with those obtained in primates.

### 4.1. Mouse rod (rhodopsin) driven IRFs and MTFs

We measured rod (rhodopsin) driven IRFs and MTFs in the LIAIS mice. As expected they were largest at low luminances. The IRFs displayed a N1 and a P1 component but with substantially longer delay times than those of L*- and S-opsin-driven IRFs (N1: 30 ms for rod-driven IRFs vs. 15–20 ms for cone-driven IRFs; P1: 60 vs. 35–45 ms for rod- and cone-driven IRFs respectively; see Figure 2). This was also found for the a-and b-waves in the mouse flash ERG (Falk et al., 2019; Ryl et al., 2021).

Interestingly, the rod-driven IRFs displayed a very prominent late negative component (N2) particularly at the lowest ML (−0.8 log cd/m^2^; Figure 2) and that could be consistently measured in individual animals (Figure 3). This component was also prominently present in luminance-driven mouse IRFs at low MLs (Stallwitz et al., 2022). It possibly is related to the STR (Saszik et al., 2002), which has been proposed to reflect ganglion cell activity (Saszik et al., 2002; Alarcon-Martinez et al., 2010; Porciatti, 2015). The latency of the N2 (122 ms at −0.8 log cd/m^2^ ML and 111 ms at −0.2 log cd/m^2^ ML) is shorter than the 200 ms latency of the negative STR and similar to the 110 ms delay of the positive STR (Saszik et al., 2002).

The rod-driven IRFs and the N2 component are easy to obtain with TWN stimuli. Furthermore, since the whole recording period is used for calculating the IRFs, opposed to time windows after a flash, and since, in contrast to flashes, no interstimulus time intervals are necessary, the N2 component can be reliably obtained with a large signal-to-noise ratio. Therefore, the N2 component may be a very interesting biomarker for retinal ganglion cell activity. The apparent latency of the rod-driven responses at low luminances, obtained from the phase plot of the MTFs, was about 82.5 ms, confirming that the MTF was to a large extent determined by the N2 component. It further was substantially larger than the apparent latencies (between 40 and 53 ms) estimated from direct ERG recordings to rod-isolating sinewaves at low luminances (Tsai et al., 2017). This indicates that homologs to the N2 component are not present in the sinewave responses. The N2 component is neither present in mouse flash ERGs. Possibly, the large contrasts used in the flash stimuli drive the N2 generating mechanisms into saturation that is not present with the subtler TWN stimuli, that resemble natural scenes more closely, and that keep the retina in a more responsive state.

### 4.2. Rod-driven IRFs show characteristics of cone IRFs at high mean luminances

Rod-driven IRFs at high MLs could be measured (Figure 2C). These IRFs resembled inverted cone-pigment-driven ERGs. Such inverted responses at high luminances were previously found to rod-isolating sinewave stimuli (Tsai et al., 2015, 2017). The origin of this response is still unclear. We cannot exclude the possibility that this response is a residual cone-driven response due to errors in the calculations of the silent substitution conditions caused by inherent assumption that had to be made. The origin of the error cannot be caused by deviations in the spectral properties of the stimulators because in the present recordings a different stimulator was used compared to the previous experiments. Errors in the estimated pigment fundamentals, caused by variability in the absorption spectra and in pre-retinal filtering, could play a role. However, the inverted cone-like rhodopsin-driven IRFs are only slightly smaller than directly measured L*-opsin-driven IRFs. It is unlikely that the error would result in a cone opsin excitation modulation that is only slightly smaller than in the direct stimulation. We therefore propose that the inverted cone-driven IRF originates in a physiological interaction between rods and cones. Rods and cones are connected through gap junctions (Ishibashi et al., 2022) that, however, involve sign conserving signal transfer whereas the inverted IRFs suggests a sign-inversion. Another possibility is a sign-inverting interaction through horizontal cells that have been described before (Szikra et al., 2014).

### 4.3. Comparison with luminance wnERGs in LIAIS mice

We correlated wnERGs obtained with opsin-isolating stimuli with luminance wnERGs in the same animals. Despite differences in mean luminances and chromaticities, luminance and L*-opsin-driven wnERGs were correlated at high MLs indicating that the luminance responses were nearly exclusively cone-driven without substantial intrusion from the rods. Rod- and luminance-driven responses at low MLs were moderately correlated with each other, again indicating that the luminance responses are mainly rod-driven. The luminance IRFs also showed the N2 component (Stallwitz et al., 2022), although less pronounced in comparison with the rod-driven IRFs.

R^2^_ML_ values describe the resemblance of wnERGs obtained at different MLs. The results indicate that rods respond best at lower luminances while cones are sensitive at higher luminances. This underlines what can already be seen in Figure 1C, where waveforms change for all single opsin-driven wnERGs with changing ML. There was little overlap in the luminance ranges where rods and cones were simultaneously sensitive, indicating the absence of a mesopic region in mice. This was also found in the responses to sinusoidal stimuli (Tsai et al., 2017). It therefore can be concluded that in mice, rod- and cone-driven responses can be obtained by using low and high MLs, respectively.

### 4.4. Comparison between L-cone photopigment driven IRFs and MTFs in mice and macaques

Cone photopigment driven wnERG measurements were recently performed in macaques (Kremers et al., 2022). The results were analyzed in a similar manner as the cone-opsin-driven wnERGs in the mice. Superficially, the L-cone-driven IRFs in mice and macaques showed similar features with an initial negative deflection N1 – possibly homolog to the flash ERG a-wave – that is followed by a positive deflection P1 – possibly homolog to the flash ERG b-wave. Oscillatory potential-like components were absent in cone-driven mouse and macaque IRFs. Similar IRFs were found for luminance stimuli in mice (Stallwitz et al., 2022), macaques (Kremers et al., 2022) and humans (Zele et al., 2017).

The L-cone-driven IRFs in macaques showed an additional positive peak (P2). In M-cone-driven IRFs the N1 and P1 components were very small but they displayed the P2 component. Kremers et al. (2022) attributed the N1 and P1 components to activity of the luminance sensitive magnocellular retino-geniculate pathway that is L-cone dominated. The P2 component was attributed to activity of the red-green color sensitive parvocellular pathway in which L-and M-cone signal strengths are more balanced. If this proposal were true then one would expect that the P2 component is absent in dichromatic mice that lack the red-green opponent pathway. This is indeed the case.

The N1 component of L^*^-opsin-driven IRFs in mice had peak times of 15–20 ms. This is similar to the peak times of the N1 delays in macaques (about 15 ms). However, the P1 components had peak times between 35 and 45 ms in the mice which is substantially larger than the P1 delay in macaques (about 20 ms). Similar delay differences could also be found in the phase plots of L*-opsin-driven MTFs: the estimated apparent delay was 37.7 ms for mice and 19.4 ms for macaques. Please observe that the delays obtained from the MTFs closely match those of the P1 components for both species. Furthermore, the delays obtained from the mice closely match the delays that were obtained in LIAIS mice with sinewave stimuli (Tsai et al., 2015, 2017). These results indicate that the apparent delays are mainly determined by the P1 component. The difference between P1 peak times in mice and macaques is a further indication that the P1 is probably homolog to the b-wave in flash ERGs, because the b-wave has longer peak times in mice compared to primates [Frishman and Wang, 2011; mice ~50 ms (Ryl et al., 2021); human ~30 ms (Hui et al., 2018); monkey ~40 ms (Bouskila et al., 2014)].

## Data availability statement

The raw data supporting the conclusions of this article will be made available by the authors, without undue reservation.

## Ethics statement

The animal study was reviewed and approved by Regierungspräsidium Mittelfranken, Ansbach, Germany.

## Author contributions

NS: planning and performance of the experiments, data analysis, interpretation of results, and writing and editing of the manuscript. AJ: conception and design of the study, planning of the experiments, data analysis, and interpretation of results. JK: conception and design of the study, construction of the stimuli, data analysis, interpretation of results, and writing and editing of the manuscript. All authors contributed to the article and approved the submitted version.

## Funding

This study was funded by the German Research Council (DFG) grant KR1317/17-1.

## Conflict of interest

The authors declare that the research was conducted in the absence of any commercial or financial relationships that could be construed as a potential conflict of interest.

## Publisher’s note

All claims expressed in this article are solely those of the authors and do not necessarily represent those of their affiliated organizations, or those of the publisher, the editors and the reviewers. Any product that may be evaluated in this article, or claim that may be made by its manufacturer, is not guaranteed or endorsed by the publisher.

## Supplementary material

SUPPLEMENTARY FIGURE 1 Correlations between cone-driven wnERGs at different MLs. Plots of the potentials obtained from L*-Opsin-driven wnERG recordings as a function of the potentials obtained at the highest ML of 1.0 log cd/m^2^. The linear regressions through the data gave the correlation coefficients r^2^_ML_ that quantified the similarity of the signals at different MLs. Correlation coefficients are shown in Figure 4D.
