# Supplementary figures and images for: Single opsin driven white noise ERGs in mice
Source: Front Neurosci. 2023 Jul 31;17:1211329. doi: 10.3389/fnins.2023.1211329 (PMC10423813; doi:10.3389/fnins.2023.1211329)

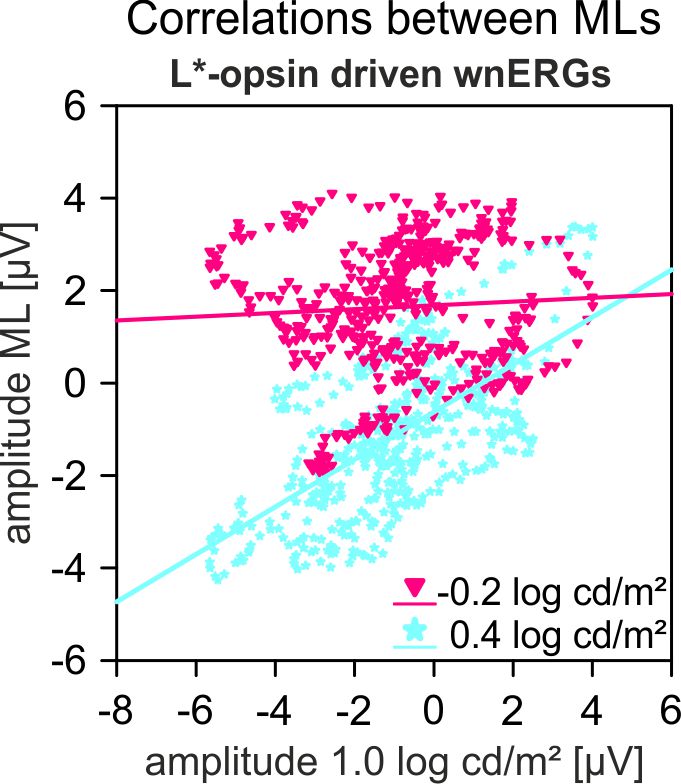

Supplement: SUPPLEMENTARY FIGURE 1 — Correlations between cone-driven wnERGs at different MLs. Plots of the potentials obtained from L*-Opsin-driven wnERG recordings as a function of the potentials obtained at the highest ML of 1.0 log cd/m2. The linear regressions through the data gave the correlation coefficients r2ML that quantified the similarity of the signals at different MLs. Correlation coefficients are shown in Figure 4D. [file Image_1.JPEG]
